# Global Patterns of Geographic Range Size in Birds

**DOI:** 10.1371/journal.pbio.0040208

**Published:** 2006-06-20

**Authors:** C. David L Orme, Richard G Davies, Valerie A Olson, Gavin H Thomas, Tzung-Su Ding, Pamela C Rasmussen, Robert S Ridgely, Ali J Stattersfield, Peter M Bennett, Ian P. F Owens, Tim M Blackburn, Kevin J Gaston

**Affiliations:** **1**Division of Biology, Imperial College London, Ascot, Berkshire, United Kingdom; **2**Department of Animal and Plant Sciences, University of Sheffield, Sheffield, United Kingdom; **3**Institute of Zoology, Zoological Society of London, Regent's Park, London, United Kingdom; **4**School of Biosciences, University of Birmingham, Edgbaston, Birmingham, United Kingdom; **5**School of Forestry and Resource Conservation, National Taiwan University, Taipei, Taiwan; **6**Michigan State University Museum and Department of Zoology, East Lansing, Michigan, United States of America; **7**Academy of Natural Sciences, Philadelphia, Pennsylvania, United States of America; **8**BirdLife International, Girton, Cambridge, United Kingdom; **9**NERC Centre for Population Biology, Imperial College London, Ascot, Berkshire, United Kingdom; The David and Lucile Packard FoundationUnited States of America

## Abstract

Large-scale patterns of spatial variation in species geographic range size are central to many fundamental questions in macroecology and conservation biology. However, the global nature of these patterns has remained contentious, since previous studies have been geographically restricted and/or based on small taxonomic groups. Here, using a database on the breeding distributions of birds, we report the first (to our knowledge) global maps of variation in species range sizes for an entire taxonomic class. We show that range area does not follow a simple latitudinal pattern. Instead, the smallest range areas are attained on islands, in mountainous areas, and largely in the southern hemisphere. In contrast, bird species richness peaks around the equator, and towards higher latitudes. Despite these profoundly different latitudinal patterns, spatially explicit models reveal a weak tendency for areas with high species richness to house species with significantly smaller median range area. Taken together, these results show that for birds many spatial patterns in range size described in geographically restricted analyses do not reflect global rules. It remains to be discovered whether global patterns in geographic range size are best interpreted in terms of geographical variation in species assemblage packing, or in the rates of speciation, extinction, and dispersal that ultimately underlie biodiversity.

## Abstract 2

The first global maps of variation in species range sizes for an entire taxonomic class--birds--reveals that range area does not follow a simple latitudinal pattern.

## Introduction

Large-scale patterns of spatial variation in species geographic range size are central to many fundamental questions in macroecology and conservation biology. These include such issues as the origin and maintenance of diversity, the potential impacts of environmental change, and the prioritisation of areas for conservation [ 1– 6]. However, the form taken by these patterns in geographic range size has remained surprisingly contentious.

Interest has focused foremost on the relationship between geographic range size and latitude, whose existence appears first to have been suggested by Lutz [ 7]. Rapoport [ 1] drew further attention to a tendency for range sizes to decline from high to low latitudes, and this was subsequently formalised as “Rapoport's rule” [ 2]. The generality of the rule has been much debated. A number of studies have argued that there is empirical evidence for the pattern [ 2, 8– 15], others that empirical evidence is lacking or is very weak [ 16– 25]. Whilst some have argued that the pattern lacks sufficient generality to be termed a “rule” [ 5, 17, 20, 24– 26], others have treated the pattern as if it were a general one or have regarded the issue as unresolved [ 3, 11, 21, 27– 31].

Latitudinal gradients in geographic range size have received such attention largely because of their possible implications for the mechanisms underlying spatial variation in species richness, and particularly the tendency for richness to be much greater in the tropics. Indeed, Stevens [ 2] argued that there may be a connection between the two. Temperate species may have larger geographic range sizes than tropical species, due to their tolerance of a broader range of environmental conditions, necessitated by greater variance in such conditions at individual sites. This could then promote higher levels of species coexistence in the tropics through a “mass effect” [ 32], whereby some of the occurrence of species is maintained by immigration of individuals into communities outside their restricted microhabitats.

Most tests for a relationship between spatial variation in species richness and geographic range size have been indirect, and based on the existence or otherwise of a latitudinal gradient in the latter. Yet, species numbers do not just vary with distance from the equator [ 33], as the recently published first map of global avian species richness clearly demonstrates [ 34]. Hotspots of bird species richness coincide with tropical mountain ranges, and richness declines away from these areas in all compass directions. If species richness and geographic range size are causally linked, then we would expect spatial variation in geographic range size to be equally richly textured. Yet, direct tests of such a relationship remain scarce, with some studies supporting a link [ 15, 35, 36] and others refuting it [ 6, 14, 18, 37].

Significantly, the majority of analyses to date of relationships both between geographic range size and latitude, and between range size and species richness, have been conducted within individual biogeographic realms. It has repeatedly been observed that the outcomes may depend on which realm or smaller biogeographic unit is being considered [ 17, 20, 22, 23, 25, 38]. In particular, studies that find evidence for the existence of Rapoport's rule are largely restricted to the Nearctic [ 5, 20], leading to the suggestion that it may be a local phenomenon that does not generalise [ 17]. Moreover, limiting analyses to individual biogeographic realms almost invariably means that species whose geographic ranges extend beyond those realms are ignored, or their ranges truncated to the limits of the realms [ 5]. The consequences for results are unknown, but may be marked where a sizeable proportion of species are distributed across multiple biogeographic realms.

What has been almost entirely missing from discussion of spatial variation in geographic range sizes has been a global perspective for major taxa. All previous studies suffer from concerns about the generality of patterns from limited numbers of biogeographic realms, and hence fail to resolve uncertainty about the true nature of spatial variation in geographic range sizes. Here, we present the first global-scale analysis of spatial variation in the geographic range sizes of species for a major taxon, all extant species of birds. We use a global database of avian distributions, mapped on an equal area projection at a scale similar to a 1° grid, to derive estimates of range sizes as the geographic breeding range area of bird species (excluding primarily marine species; see Materials and Methods). Global patterns of bird species richness were previously determined using this database [ 34], which allows us to explore in detail both spatial variation in geographic range size and its link to species richness, using data of unparalleled geographic extent and resolution.

## Results

### Species-Range Area Distributions

**Figure 1:**
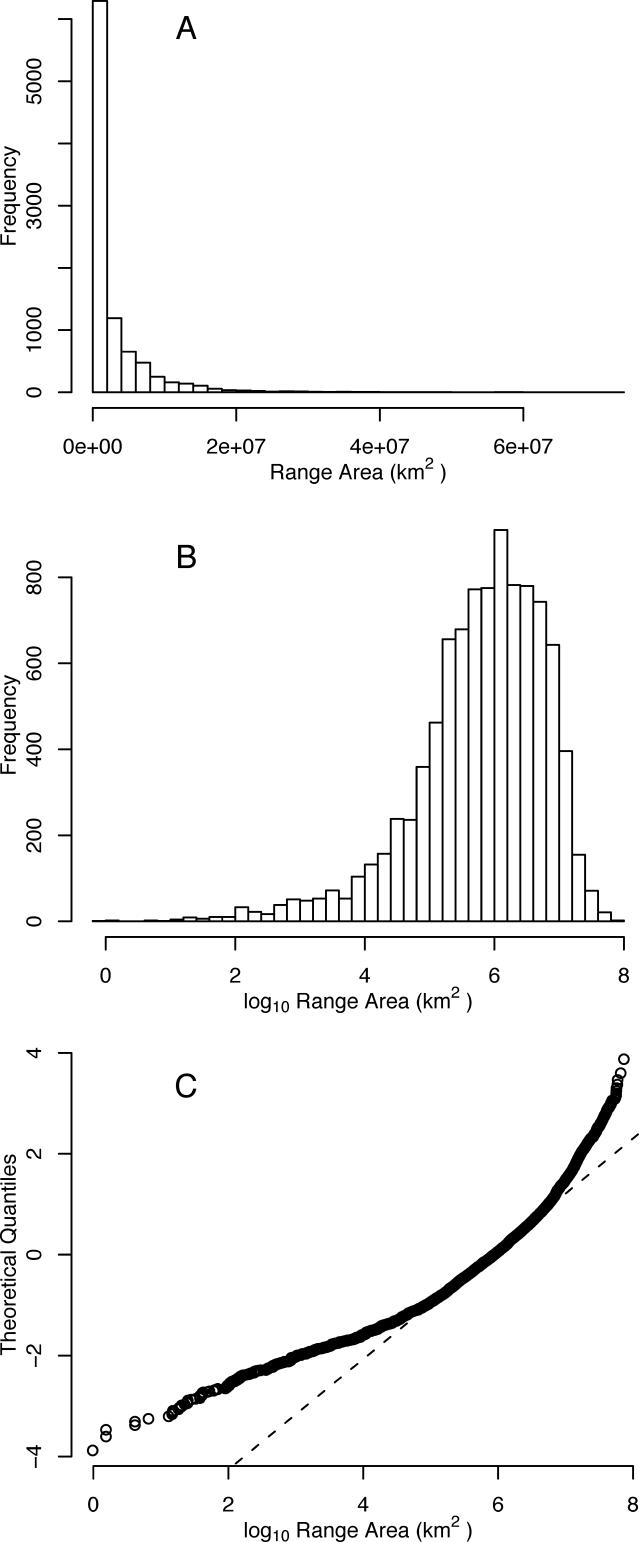
Species-Range Area Distribution for the Global Avifauna (A) Untransformed range areas. (B) Log _10_-transformed range areas. (C) Normal probability plot for log _10_-transformed range areas, showing the expectation under a normal distribution (dashed line) and the observed distribution (open circles).

Figure 1 summarizes the global species-range area distribution for birds. The distribution is strongly right-skewed ( Figure 1A), with the mean range area (2.82 × 10 ^6^ km ^2^) of the 9,505 extant species markedly larger than the median (0.87 × 10 ^6^ km ^2^). More than a quarter (27.6%) of bird species have geographic range areas smaller than 225,000 km ^2^, less than the area of Great Britain or Minnesota. The species-range area distribution is formally neither lognormal ( Figure 1B; D = 0.0656, *p* ≪ 0.001) nor logit-normal (D = 0.0636, *p* ≪ 0.001), with strong left-skew in both cases. Normal probability plots reveal that the geographic range areas of the more restricted and the more widespread species are small relative to expectation ( Figure 1C).

### Spatial Variation in Range Area

**Figure 2:**
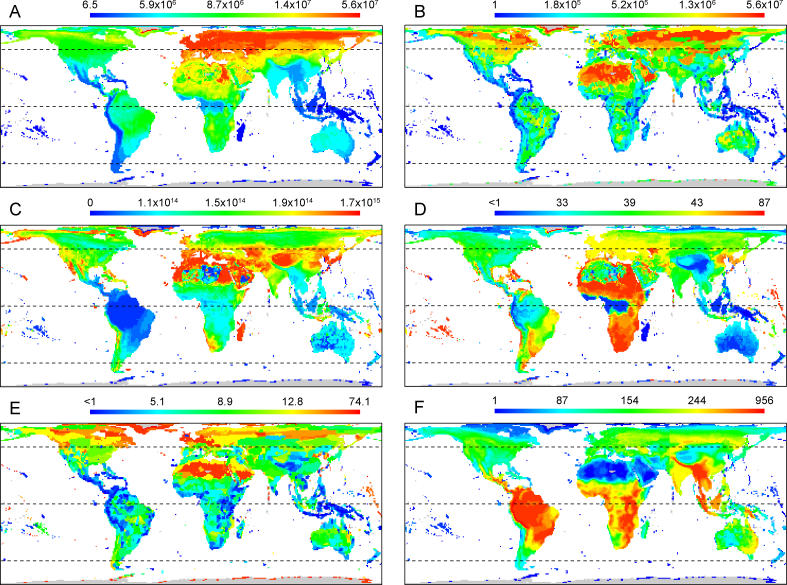
Geographic Distribution of Geographic Range Areas, Latitudinal Range Extent, and Species Richness for the Global Avifauna (A) Median geographic range area (km ^2^). (B) Minimum geographic range area (km ^2^). (C) Variance in geographic range area (km ^2^). (D) Median latitudinal range extent (degrees). (E) Minimum latitudinal range extent (degrees). (F) Total species richness. The map scales are based on quartiles of the underlying distributions; the scale bars show the quartile values for each map. Parallels are shown at 45° S, the Equator, and 45° N.

Key spatial patterns in species geographic range areas are shown in Figure 2. The median geographic range area of the species coexisting in each grid cell shows strong spatial patterns ( Figure 2A). The smallest median range areas occur on islands, in low-latitude mountainous areas, and to a large extent in the southern hemisphere ( Figure 2A), as are the smallest range areas overall (the range area of the most narrowly distributed species in each grid cell; Figure 2B). The variance in range area is typically higher in the northern hemisphere, especially in mid-latitudes ( Figure 2C). Some previous analyses of spatial variation in geographic ranges have focused on the latitudinal extent of the distributions of species. The median latitudinal extent of the bird species coexisting in each grid cell shows a rather different, but equally complex, picture to that for range area ( Figure 2D). The main differences lie in the higher Afrotropical and lower Palearctic latitudinal extents compared to the patterns of range area, however there are similarities in the distribution of the smallest median extents, notably in mountainous regions and some island groups. The spatial distribution of the smallest latitudinal extents (the range extent of the most narrowly distributed species in each grid cell; Figure 2E) is very similar to that of the smallest range areas ( Figure 2B).

**Figure 3:**
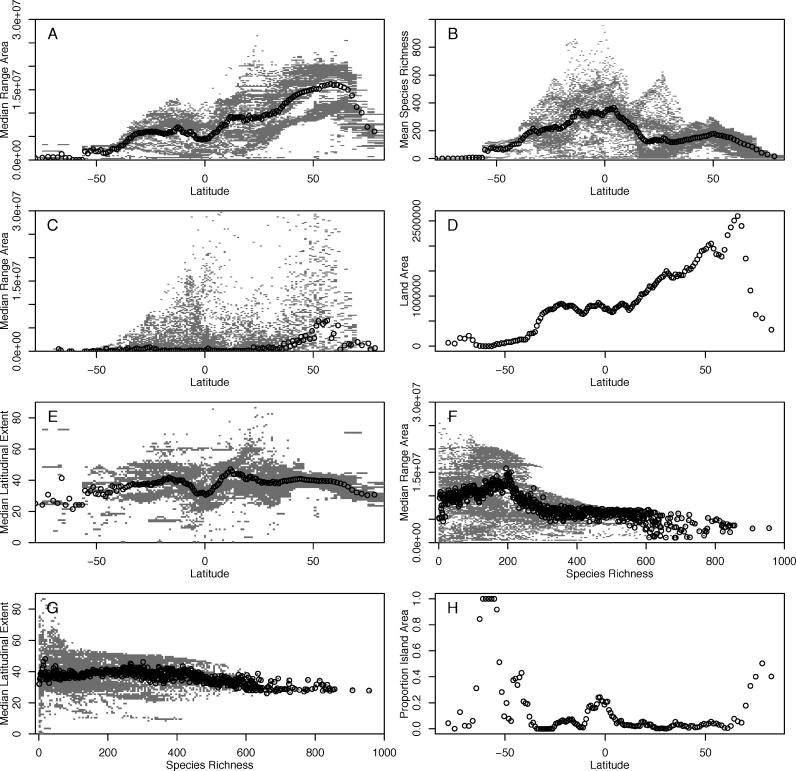
Global Relationships between Geographic Range Area, Latitudinal Range Extent, Species Richness, Land Area, Island Area, and Latitude (A) Median geographic range area and latitude. (B) Species richness and latitude. (C) Median range area and latitude for species with midpoints falling in each respective latitudinal band. (D) Total land area (km ^2^) within latitudinal bands. (E) Median latitudinal range extent and latitude. (F) Median geographic range area and species richness. (G) Median latitudinal range extent and species richness. (H) Proportion island area and latitude. For (A–C) and (E and F), open circles represent latitudinal means, and grey points show the spread of individual grid cell values. Southern latitudes are indicated as negative, northern ones as positive.

The latitudinal trends in species geographic range sizes are summarized in Figure 3. Contrary to the predictions of Rapoport's Rule, geographic range area does not decline towards the tropics in both hemispheres ( Figure 2A, Table 1). Rather, median range area is greatest at high, albeit not the highest, northern latitudes and decreases toward high southern latitudes. This relationship shows a subtle change in the magnitude of the slope at the equator, but does not show the change in sign that Rapoport's rule would predict ( Figure 3A; scoring southern latitudes as negative, range size versus latitude across all cells: *r* = 0.60, *n* = 17,867, *p* ≪ 0.001; for cell averages per latitudinal band: *r* = 0.89, *n* = 151, *p* ≪ 0.001). There is no relationship between median range area and absolute latitude (i.e. regardless of hemisphere), once the sample size dominance of the northern hemisphere has been removed by averaging within latitudinal bands (across all cells: *r* = 0.45, *n* = 17,867, *p* ≪ 0.001; cell averages per latitudinal band: *r* = 0.07, *n* = 151, *p* = 0.40).

**Table 1:**
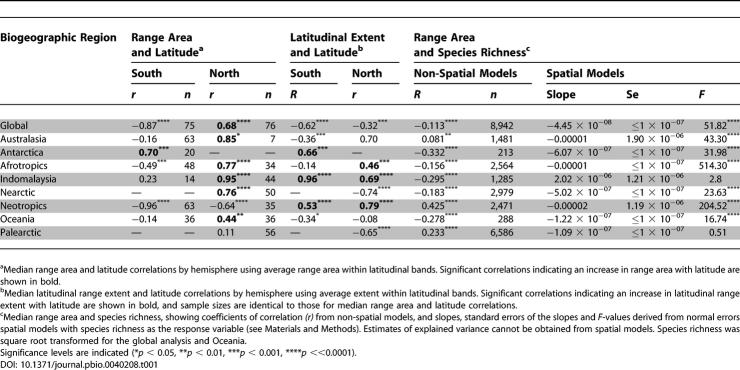
Global and Within-Realm Patterns in Range Size

Within individual biogeographic realms and hemispheres, median range areas increase with latitude in only seven out of 13 cases, and six out of these seven are in the northern hemisphere ( Table 1; see Protocol S2 for tests using all cells). In contrast, species richness shows strong latitudinal correlations with highest richness in tropical regions, and notable peaks in the Andes, Himalayas and African Rift Valley ( Figure 2F; richness [square root transformed] versus latitude across all cells: southern hemisphere *r* = −0.56, *n* = 4,903, *p* ≪ 0.001, northern hemisphere *r* = −0.39, *n* = 12,964, *p* ≪ 0.001; richness versus latitude for cell averages per latitudinal band: southern hemisphere *r* = −0.98, *n* = 75, *p* ≪ 0.001, northern hemisphere *r* = −0.77, *n* = 76, *p* ≪ 0.001). In addition, the southern hemisphere shows both higher diversity at mid-low latitudes and a steeper decline with increasing latitude than the northern hemisphere ( Figure 3B). These analyses are not sensitive either to the omission of island cells or to more conservative tests using differences in latitudinal values that account for latitudinal autocorrelation (see Protocol S2).

The relationship between geographic range area and latitude is not an artefact of the way that geographic range size variation was quantified. Because analyses based on the range size characteristics of species in cells can be biased by shared species composition, we also checked the relationship using the “midpoint method” [ 16]. In this method, species are allocated to latitudinal bins based on the location of the latitudinal mid-points of their geographic ranges, so each species only contributes to one data point, and the median range area of the species in each bin is plotted against the latitude of that bin ( Figure 3C). Globally, these data show an increase in median geographic range area from southern to northern latitudes ( *r* = 0.58, *n* = 126, *p* ≪ 0.001) and the decline in range areas at high northern latitudes is more marked using the midpoint method. Within hemispheres, there is no global trend in southern latitudes and a strong increase in northern latitudes; within hemispheres and realms, median geographic range areas increase significantly with latitude in only three out of 13 cases (see Protocol S2). Although phylogenetic autocorrelation could influence these findings, evidence suggests that species range area shows low phylogenetic dependence (see Protocol S2; [ 5, 39]) and thus such autocorrelation will have a weak effect.

The latitudinal gradient in land area across all latitudes ( Figure 3D) is strongly correlated with median range area ( Figure 3A, *r* = 0.72, *n* = 152, *p* ≪ 0.0001), particularly if the sharp decline in both variables above 67° N is omitted ( *r* = 0.95, *n* = 146, *p* ≪ 0.0001). One possible explanation of the patterns in range area is thus that the latitudinal extents of ranges ( Figure 2D) increase from low to high latitudes but that the longitudinal constraints on range area mask this relationship. However, the latitudinal gradient in species latitudinal range extents ( Figure 3E) shows weaker support for Rapoport's rule than does that in range area. Globally, latitudinal extent decreases, rather than increases, from low to high latitudes in both hemispheres ( Table 1). Within individual biogeographic realms and hemispheres, latitudinal extent increases with latitude in only six out of 13 cases ( Table 1). Indeed, in the northern hemisphere, increases in land area seem to mask decreases in latitudinal extent at higher latitudes.

### Range Area and Species Richness

Simple correlations between median geographic range area and species richness ( Figure 3F) yield significant negative relationships globally and positive relationships in three out of eight biogeographic realms ( Table 1). A more consistent picture is revealed once the similarity of proximal cells arising from spatial autocorrelation is explicitly modelled. This method shows that at a global scale median range area and species richness are negatively related ( Table 1). Also, when individual biogeographic realms are considered separately, there is a negative relationship between range area and species richness in seven of the eight realms, with the only positive relationship being statistically non-significant ( Table 1).

## Discussion

The global species-range area distribution is strongly right-skewed, with the majority of species having small, but not the smallest, geographic ranges ( Figure 1). This is consistent with previous studies, both of birds and other taxa, within biogeographic realms [ 5, 6, 40– 43]. The departure from a log-normal and a logit-normal distribution, the two null models that have previously been suggested, is also consistent with the findings of such studies [ 5, 43]. Such departures are typically interpreted as resulting from a lack or an excess of rare species relative to expectation [ 6, 44, 45]; normal probability plots ( Figure 1C) show that here both for the log-normal and a logit-normal the range areas of the more widespread species are also small relative to expectation. This is likely to be a consequence of dispersal limitation of species to a subset of the major land masses as even the most widespread species are not cosmopolitan.

Overall, there is no global tendency for avian geographic range sizes to decline in area, or in latitudinal extent, towards the tropics ( Figures 2A, 2D). Rather, there is a general trend of declining median range area from high northern latitudes to high southern ones ( Figure 3A). This leads to entirely different relationships between range area and latitude in different biogeographic realms, with those in the northern hemisphere typically conforming to Rapoport's rule, and those in the southern hemisphere failing to do so ( Table 1). This both confirms that Rapoport's rule does not generalise [ 17, 20, 46], and cautions against assuming that biological patterns from the relatively well-studied northern temperate regions will apply to the rest of the world [ 47]. Current evidence suggests that Rapoport's rule in other taxa is limited principally to the same regions as for birds [ 20]. Nevertheless, we have shown here that a global perspective is needed truly to understand spatial variation in geographic range sizes, as conclusions based on single/limited biogeographical data may not be informative of overall pattern. It will be interesting to test the wider generality of the relationships shown for birds, as data for other taxa become available.

Description of variation in avian geographic range areas in terms of latitude alone masks substantial spatial patterning, especially at low latitudes and in southern regions ( Figure 2A). Small range areas are associated, perhaps unsurprisingly, with islands, but also with mountain ranges in the tropics and sub-tropics. This suggests that range areas may be constrained by the availability of land area within the climatic zones to which species are best adapted [ 48]. Thus, in South America, for example, the relatively climatically uniform expanse of the tropical lowland Amazon basin allows broad geographic ranges in species evolving within this region, in contrast to the restricted ranges available to species evolving to exploit any of the restricted climatic zones that pertain at different altitudes in the tropical Andes. This idea is also consistent with the global decline in median geographic range areas from high northern to high southern latitudes being generated by the availability of land area in different climatic zones. The overall decline corresponds well with the latitudinal pattern of land area ( Figure 3D). Additionally, regions with particularly small median range area correspond well with regions where the proportion of land area contributed by islands is high ( Figure 3H), although this latter pattern is not sufficient of itself to generate the latitudinal gradient in range area (see Protocol S2).

The evident contrast between the global decline in median range area from high northern to high southern latitudes and the equatorial peak in species richness argues against any simple relationship between geographic range area and species richness ( Figure 3F; or between latitudinal range extent and species richness: Figure 3G). However, whilst the complexities of the patterns of spatial variation in both variables mean that using non-spatial models may be problematic, there is nonetheless a significant (albeit weak) negative global relationship between the two ( Table 1). This is echoed by weak negative relationships in five of the biogeographic realms separately, with a significant positive relationship between range size and species richness, contrary to Rapoport's rule, in the other three ( Table 1). Nevertheless, relationships between richness and range area are likely to be heavily influenced by spatial autocorrelation, since neighbouring areas tend to contain largely the same numbers and average range sizes of species, and so do not contribute independent information to tests of association.

Using spatial models, therefore, a rather different picture emerges. Globally, median range area and species richness remain significantly negatively related, as they do in four of the biogeographic realms ( Table 1). However, within one region, Indomalaysia, a previously significant negative relationship becomes non-significant using a spatial model, and within two regions, the Neotropics and Australasia, significant positive correlations become significant negative relationships after controlling for spatial autocorrelation. In these last two regions, numerous grid cells falling within the Amazon basin and central Australian deserts dominate simple models. However, the similarity in species composition between these cells, as a consequence of the large range areas of the species occupying them, means that spatial autocorrelation is high across these areas. Their influence is dampened in spatial models in favour of less well-represented and less pseudo-replicated areas of high topographic variability and species richness but low geographic range area in the mountains of the Andes and Great Dividing Range. Thus, the fact that the richest areas house species with the smallest mean range areas was obscured by the numerical dominance of pseudo-replicated areas of lower richness housing species with broad geographic distributions. This emphasizes the potential importance of a spatially-explicit perspective in understanding large-scale biodiversity patterns, an approach that has almost been entirely lacking in previous considerations of spatial variations in geographic range size. It also suggests that whilst at a global scale for birds there is little support for Stevens' [ 2] notion of a general latitudinal gradient in range size, there is nonetheless some support for the link he suggested between geographic range size and species richness.

We end with two notes of caution regarding the ecological and evolutionary mechanisms that may underlie the relationship between geographic range size and species richness. First, although we have detected statistically significant associations between range area and species richness, the strength of these relationships is typically not strong. For those analyses based on non-spatial models the maximum proportion of variance explained was only 18% ( Table 1), and for spatial models of the form employed here explained variances cannot be derived (the shallow slopes reflect the relative magnitudes of the two variables). It would thus be wrong to conclude that smaller range areas are strongly associated with higher levels of species richness, although there is robust evidence that the two variables are not entirely independent, both globally and within some biogeographic realms. Second, so far we have largely discussed the link between range area and species in the context of Stevens' [ 2] mass effect mechanism, but it is equally plausible that other mechanisms could underlie the same pattern. It has been suggested, for instance, that spatial patterns in geographic range size may be due to geographical variation in the processes of speciation and extinction that ultimately generate biodiversity [ 5, 49]. Although recent studies have confirmed that there is geographic variation in the net rate of cladogenesis [ 5, 50, 51], the relative role of such phenomena in determining large-scale patterns of diversity remains to be discovered.

## Materials and Methods

### Data.

The analyses presented here are based on a previously reported database [ 34] of distribution maps for 9,505 extant, recognized bird species following a standard avian taxonomy [ 52]. This excludes primarily marine species, the vast majority of the geographic ranges of which are oceanic and thus of quite different character, and for which comparable data are not available. Primarily marine species were defined as those regularly foraging more than 50 km from land during the breeding season (and thus for which land did not constitute a high proportion of their ranges), were identified using a variety of sources [ 53– 58], and were excluded from the analyses presented here. To obtain information on the geographical breeding range data on a global scale we used a wide range of data sources [ 34], which are described in detail in Protocol S1 along with the associated notes on methods and availability. Briefly, breeding ranges from the published sources were mapped as vectors or “polygons” and converted to an equal area grid for analysis. The grid used a Behrmann projection and a cell size (96.3 km) that gives a scale identical to 1° grids at the 30° latitude of true scale. The vertical cell boundaries coincide with 1° lines of longitude but the horizontal boundaries vary systematically in their latitudinal separation, giving a grid with 360 columns and 152 rows. We have used these 152 equal area longitudinal bands for calculating latitudinal averages and for binning species by their latitudinal range midpoint. Species were scored as present in a grid cell if any of the available vector sources suggested that the breeding range fell within the cell boundaries. Overall species richness was derived by summing all species present within each cell. Cell land areas were calculated using a coastline vector dataset [ 59] divided into continental and island land masses. The geographic range areas of individual species were estimated as the sum of the areas of the cells in which they were scored as occurring. This will tend disproportionately to overestimate the range areas of particularly narrowly distributed species, and those whose distributions are associated with linear features (e.g. mountain chains, rivers), but this is unlikely to influence the broad patterns reported here. Latitudinal extent was defined as the difference between the northern and southern limits of the vector maps showing each species' breeding range. Biogeographic realms were delimited using the World Wildlife Fund ecoregions map [ 60].

### Analyses.

To calculate the logit transformation of geographic range area we used the global breeding area of all species as the upper limit to the occurrence of any individual species [ 43]. The shapes of logarithmically- or logit-transformed range area distributions were assessed using Kolmogorov-Smirnov tests of the transformed data against normal distributions with mean and standard deviation taken from the transformed data. Species richness was untransformed or square root transformed for analyses, as appropriate, better to normalise distributions for particular tests. Relationships between median geographic range area/latitudinal extent or species richness and latitude were determined using correlation coefficients. Relationships between median geographic range area/latitudinal extent and species richness were determined using correlation coefficients, and using normal errors generalised least squares (GLS) models (SAS; 62), fitting spherical spatial covariance structures with longitudinal and latitudinal cell centroid values as spatial variables, in SAS version 9.1.3. GLS models took account of the differences among major biogeographical realms, in the maximum geographic distance or range parameter (ρ), measured in degrees, over which spatial autocorrelation in equivalent independent errors model residuals was observed to occur. This involved estimating ρ from the semi-variogram of residuals of non-spatial normal errors models that included the relevant combination of predictors, separately for each realm. All eight estimates of ρ were then entered as spatial covariance parameters in the model, with spatial autocorrelation assumed for observations within the same realm. Global models were run on a regular 50% grid of cells (chequerboard) in the full data set due to computer memory constraints (even when run on a mainframe); tests on regional subsets of the data confirmed that use of the reduced data set would not alter the conclusions.

## Supporting Information

Protocol S1 Data Sources and Data Availability (90 KB DOC)

Protocol S2 Results of Analyses of Effects of Islands; Differencing of Relationships with Latitude; and Phylogenetic Autocorrelation of Geographic Range Area (46 KB DOC)
