# Supplementary material for: Global Patterns of Geographic Range Size in Birds
Source: PLoS Biol. 2006 Jun 20;4(7):e208. doi: 10.1371/journal.pbio.0040208 (PMC1479698; doi:10.1371/journal.pbio.0040208)
Supplement: Supplementary file 1 [file pbio.0040208.sd001.doc]

**Protocol S1**

**(i) Data Sources**

As reported previously [1], we used a wide range of data sources to provide information on the geographical breeding ranges of bird species [2-35] and collated these data to form a single electronic database with each species represented by one or more polygons (Supplementary Table 1). Because our aim was to build a global database of breeding ranges, we preferentially used data sources that provided information on breeding ranges over large geographical areas and for a taxonomically diverse set of species where these were available, rather than sources restricted to a small number of species or a small geographical area. We also made preferential use of sources according to the format of data they contained, with our preference order being (from high to low preference): Electronic polygons (that could be imported directly into our polygon database); Range maps (that could be scanned, digitized and converted into polygons); Atlas data in the form of a presence/absence grid for each species (that could be imported as an electronic grid for each species and then converted into polygons); Presence/absence data on the occurrence of species on small islands (which could be converted to polygons covering the entire island in question – this approach was only used for islands whose total area was much smaller than the resolution of our final analyses); Text description of geographical breeding range (which were drawn by hand as a polygon using a digitizing tablet). So, for instance, if we were able to obtain data on the full geographical range of a species in the form of electronic polygons we did not seek additional information on that species. Polygons derived from each source were stored separately so that we could establish the origin of all information.

This approach of preferentially using data sources that cover large geographical areas and large numbers of sources is different from that adopted for more localized databases, which typically make greater use of data sources that give information from single point localities and on single species. The reason we adopted our approach is that the fine-scale approach is as yet impractical at the global scale. It can be shown for restricted areas, however, that the two approaches produce highly congruent pictures of large-scale distribution, with a correlation between species richness values derived from the alternative methods of 0.96 for the sub-Saharan African region [1].

**(ii) Data availability**

The global polygon database described and analysed here was collated as part of a NERC Consortium Grant project, but includes data that were collected by a wide variety of persons and organisations with diverse interests. With respect to inspecting data quality, all of these sources [2-35] are publicly available either as electronic sources or in the published literature so it is possible to view the exact information used in this study (see Supplementary Table 1 for detailed list of sources and associated notes). Indeed, some of the data sources have already been made publicly available as electronic polygons [19, 32]. In other cases, however, the original data providers remain the owners of these data and, although we have permission to use their data in our analyses, we do not have permission to publicly distribute the original data. In addition, some of these data are considered by the owners to be of commercial interest. It is therefore not possible to make the full database publicly available in an electronic form at the present time. However, those interested in academic research should contact I.P.F. Owens at Imperial College London ([i.owens@imperial.ac.uk](mailto:i.owens@imperial.ac.uk)) with requests regarding data availability. Also, the consortium will make publicly available those geographical data collected as part of the consortium grant that are under the ownership of the principal investigator within two years of the end of the consortium grant, meaning that these data will be available from the end of March 2009.

1. Orme CDL, Davies RG, Burgess M, Eigenbrod F, Pickup N, Olson V, Webster AJ, Ding T-S, Rasmussen PC, Ridgely RS, Stattersfield AJ, Bennett PM, Blackburn TM, Gaston KJ, Owens IPF (2005) Biodiversity hotspots of species richness, threat and endemism are not congruent. Nature 436: 1016-1019.
2. BirdLife International (2000) Threatened birds of the world Barcelona and Cambridge: Lynx Edicions and BirdLife International.
3. Hazevoet CJ (1995) The birds of Cape Verde London: British Ornithological Union.
4. Jones P, Tye A (2003) The birds of Principe, Sao Tome and Annobon London: British Ornithological Union.
5. Langrand O (1990) Guide to the birds of Madagascar Austin: Yale University.
6. Rowlands B (1998) The birds of St Helena London: British Ornithological Union
7. Sinclair I, Langrand O (1998) Birds of the Indian Ocean Islands Cape Town: Struik Publishers.
8. Cramp S, Snow D, Perrins CM (1997) The birds of the Western Palearctic: Concise Edition. Vols 1 & 2. Oxford: Oxford University Press.
9. Brown L, Urban E, Newman K (eds) (1982) The birds of Africa: Vol 1 London: Academic Press.
10. Urban E, Fry C, Keith S (eds) (1982) The birds of Africa: Vol 2 London: Academic Press.
11. Fry C, Keith S, Urban E (eds) (1988) The birds of Africa: Vol 3 London: Academic Press.
12. Keith S, Fry C (eds) (1992) The birds of Africa: Vol 4 London: Academic Press.
13. Urban E, Keith S, Fry C (eds) (1997) The birds of Africa: Vol 5 London: Academic Press.
14. Fry C, Keith S (eds) (2000) The birds of Africa: Vol 6 London: Academic Press.
15. Fry C, Keith S, Urban E (eds) (2004) The birds of Africa: Vol 7 London: Academic Press.
16. Porter RF, Christensen S, Schiermacker-Hansen P (1996) Field guide to the birds of the Middle East London: Poyser.
17. Rasmussen P. C., Anderton J (2005) Birds of South Asia. The Ripley Guide. Vols 1& 2. Washington DC and Barcelona: Smithsonian Institution and Lynx Edicions.
18. Flint VE, Boehme RL, Kostin YV, Kuznetsov AA (1984) A field guide to the birds of the USSR Princeton: Princeton University Press.
19. Ding T.-S (2005) Distribution of breeding birds in East Asia (<http://asianbird.zo.ntu.edu.tw/>) Taiwan: National University of Taiwan.
20. Bull PC, Gaze PD, Robertson CJR (1985) The atlas of bird distribution in New Zealand The Ornithological Society of New Zealand.
21. Pratt HD, Bruner PL, Berrett DG (1987) The birds of Hawaii and the Tropical Pacific Princeton.
22. Beehler B.M, Pratt TK, Zimmerman DA (1986) Birds of New Guinea New Jersey: Princeton University Press.
23. Bregulla HL (1992) Birds of Vanuatu Anthony Nelson.
24. Coates BJ (1985) The birds of Papua New Guinea: Volume 1 Queensland: Dove Publications.
25. Coates BJ (1990) The birds of Papua New Guinea: Volume 2 Queensland: Dove Publications.
26. Doughty C, Day N, Plant A (1999) Birds of Solomons, Vanuatu & New Caledonia London: Christopher Helm.
27. Mayr, E., Diamond, J. (2001) The birds of northern Melanesia. Oxford: Oxford University Press.
28. Strange MA (2001) Photographic guide to the birds of Indonesia Princeton: Princeton University Press.
29. Barrett G, Silcocks A, Barry S, Cunningham R, Poulter R (2003) The new atlas of Australian birds Victoria: Royal Australasian Ornithologists Union.
30. Shirihai H (2002) The complete guide to Antarctic wildlife: birds and marine mammals of the Antarctic Continent and the Southern Ocean Princeton: Princeton University Press.
31. Marchant S, Higgins PJ (eds) (1990) Handbook of Australian, New Zealand and Antarctic birds. Vol 1. Parts A and B. Melbourne: Oxford University Press.
32. Ridgley RS et al. (2003) Digital distribution maps of the Western Hemisphere. Version 1.0. Arlington, Virginia: NatureServe.
33. Heinzel H, Hall B (2000) Galapagos Diary - A complete guide to the archipelago's birdlife California: University of California Press, California.
34. National Geographic (1999) Field guide to the birds of North America. Washington, D.C.: National Geographic.
35. Sibley CG, Monroe BL (1990) Distribution and taxonomy of the birds of the worlds New Haven: Yale University Press.

**Supplementary Table 1.** Breakdown of data sources by biogeographical realm. Sources for geographical range data are shown along with the method by which those data were converted into polygons: E – source provides data as polygons; I – polygons created for entire islands only using presence/absence information in source; D – polygons digitized from scanned range maps in source; G – polygons created by converting atlas grid records in source; T – polygons created from text descriptions of geographic range in source. The number of species used from each source is also shown. Footnotes are used to provide finer grained detail of the areas used for each source within realms. The Birds of Africa source covers maps from all seven volumes [Refs 9-15].

|  | Biogeographic realm | | | | | | | |  |
| --- | --- | --- | --- | --- | --- | --- | --- | --- | --- |
| Data source | Afrotropics | Palearctic | Indomalaysia | Oceania | Australasia | Antarctica | Neotropics | Nearctic | No of Species |
| BirdLife International, 2002 | E^1^ | E^1^ | E^1^ | E^1^ | E^1^ | E^1^ | E^1^ | E^1^ | 1338 |
| Hazevoet, 1995 | I^2^ |  |  |  |  |  |  |  | 34 |
| Jones and Tye, 2003 | I^3^ |  |  |  |  |  |  |  | 203 |
| Langrand, 1990 | D^4^ |  |  |  |  |  |  |  | 195 |
| Rowlands, 1998 | I^5^ |  |  |  |  |  |  |  | 54 |
| Sinclair and Langrand, 1998 | I^6^ |  |  |  |  |  |  |  | 105 |
| Cramp et al., 1997 | D^7^ | D^8^ |  |  |  |  |  |  | 610 |
| Birds of Africa, 1982 - 2004 | D^9^ | D^10^ |  |  |  |  |  |  | 2117 |
| Porter et al., 1996 | D^11^ | D^11^ |  |  |  |  |  |  | 462 |
| Rasmussen & Anderton, 2005 |  | G^12^ | G^12^ |  |  |  |  |  | 507 |
| Flint et al., 1984 |  | D^13^ | D^13^ |  |  |  |  |  | 637 |
| Ding, 2005 |  | E^14^ | E^1^ | E^15^ | E^16^ |  |  |  | 2400 |
| Bull et al., 1985 |  |  |  | G^17^ | G^18^ |  |  |  | 230 |
| Pratt et al., 1987 |  |  |  | I^19^ |  |  |  |  | 437 |
| Beehler et al., 1986 |  |  |  |  | T^20^ |  |  |  | 24 |
| Bregulla, 1992 |  |  |  |  | I^21^ |  |  |  | 74 |
| Coates, 1985 and 1990 |  |  |  |  | D^20^ |  |  |  | 727 |
| Doughty et al., 1999 |  |  |  |  | I^22^ |  |  |  | 293 |
| Mayr and Diamond, 2001 |  |  |  |  | I^23^ |  |  |  | 287 |
| Strange, 2001 |  |  |  |  | D^24^ |  |  |  | 267 |
| Barrett et al., 2003 |  |  |  |  | G^25^ | G^26^ |  |  | 782 |
| Shirihai, 2002 |  |  |  |  | I^26^ | I^26^ |  |  | 147 |
| Marchant and Higgins, 1990 |  |  |  |  |  | D^27^ |  |  | 17 |
| Ridgley et al., 2003 |  |  |  |  |  |  | E^1^ | E^1^ | 4086 |
| Heinzel and Hall, 2000 |  |  |  |  |  |  | I^28^ |  | 120 |
| National Geographic, 1999 |  |  |  |  |  |  |  | D^29^ | 47 |
| Sibley and Monroe, 1990 | T^1^ | T^1^ | T^1^ | T^1^ | T^1^ |  | T^1^ |  | 26 |

Notes: 1 – Source covers entire realm; 2 – Cape Verde only; 3 – Sao Tome, Principe and Annabon only; 4 – Madagascar only; 5 – St. Helena only; 6 – Indian Ocean islands only; 7 – Northern Afrotropics only; 8 – Western Palearctic only; 9 – Continental Africa only; 10 – North Africa only; 11 – Middle East region only; 12 – Afghanistan, Pakistan and India only; 13 – Former USSR only; 14 – Eastern Palearctic to 70° E only; 15 – Western Oceania islands only; 16 – West of ‘Lydekker Line’ only; 17 – New Zealand islands only; 18 – Southwest Oceania islands only; 19 – Hawaii archipelago and tropical Pacific islands only; 20 – Papua New Guinea & Bismark archipelago only; 21 – Vanuatu archipelago only; 22 – Solomons, Vanuatu and New Caledonia archipelagos only; 23 – Northern Melanesia only; 24 – Irian Jaya only; 25 – Australia only; 26 – Southern Ocean islands only; 27 – Continental Antarctica only; 28 – Galapagos archipelago only; 29 – Greenland only
