# Supplementary material for: Global Patterns of Geographic Range Size in Birds
Source: PLoS Biol. 2006 Jun 20;4(7):e208. doi: 10.1371/journal.pbio.0040208 (PMC1479698; doi:10.1371/journal.pbio.0040208)
Supplement: Supplementary file 2 [file pbio.0040208.sd002.doc]

**Protocol S2**

**(i) Islands**

A potential confounding factor in analyses of geographic range size and latitude is the greater prevalence of islands in the southern hemisphere, since island dwelling may restrict range size [1]. In the southern hemisphere, 18% of grid cells contain only island land area (1183/6448), compared to 13% in the northern hemisphere (1695/13111). Omitting island cells from the calculation of latitudinal correlations in median range area and species richness does not, however, qualitatively change our results. Median range area shows a significant positive correlation with latitude across all latitudes (across all cells: r=0.63, n=15183, p<<0.001; cell averages per latitudinal band: r=0.90, n=146, p<<0.001). The correlations within hemispheres are consistent with analyses using the full data set (Supplementary Table 2): southern hemisphere (across all cells: r=-0.58, n=3831, p<<0.001; cell averages per latitudinal band: r=-0.94, n=70, p<<0.001); northern hemisphere (across all cells: r=0.46, n=11352, p<<0.001; cell averages per latitudinal band: r=0.67, n=76, p<<0.001). The correlations between richness and latitude are also consistent with analyses including island cells (across all cells: southern hemisphere r=-0.67, n=3831, p<<0.001, northern hemisphere r=-0.45, n=11352, p<<0.001; cell averages per latitudinal band: southern hemisphere r=-0.98, n=70, p<<0.001, northern hemisphere r=-0.77, n=76, p<<0.001).

**(ii) Differencing**

The results of latitudinal correlations were also validated using differencing to control for possible effects of spatial autocorrelation [2]. Differencing is a highly conservative test assuming that variables follow a first-order autoregressive process, with the true behaviour of empirical data likely to lie between this scenario and that of an independent errors test (e.g. a correlation coefficient). The first differences between adjacent points were taken, north minus south, and tested with Student’s *t* against a mean difference of zero. Such differenced data effectively detrends latitudinal gradients, showing average changes with latitude. Latitudinal differences in median range area difference suggest no overall mean change (t=-0.88, n=150, NS) but this is driven largely by the sharp drop in range size above 65° N. Omitting these points shows a consistent overall mean decrease from north to south across both hemispheres (t=-3.67, n=143, p < 0.001). In contrast, differences in species richness show a significant mean decrease away from the equator in the southern hemisphere (t=-3.23, n=75, p<0.01) and a significant mean increase toward the equator in the northern hemisphere (t=3.97, n=75, p<<0.001).

**(iii) Phylogenetic autocorrelation**

Standard comparative tools for addressing phylogenetic autocorrelation are not readily applicable in these analyses because the units of analysis are geographic cells rather than species. The cell values do, however, derive from species values and thus the data may contain signals of phylogeny and, indeed, the interaction of phylogeny and space. Methods for simultaneously addressing spatial and phylogenetic autocorrelation are in their infancy and would require more resolved and complete species level phylogenies than are currently available for birds. However, such concerns are mitigated because the variables concerned show weak phylogenetic dependence. First, variance components analysis [3] shows that 81% of variation in species range area is at the genus level or below, with the majority of this (64%) at the species level: thus, relatively little variation is found within the higher levels of orders (1%) and families (18%). Second, an assessment of phylogenetic dependence [4] using family mean range areas and a family level tree [5] suggests low phylogenetic dependence (maximum likelihood estimate of λ = 0.038, 95% confidence limits 0 – 0.33).

1. Manne LL, Brooks TM, Pimm SL (1999) Relative risk of extinction of passerine birds on continents and islands. Nature 399: 258–261.
2. Koleff P, Lennon JJ, Gaston KJ (2003) Are there latitudinal gradients in species turnover? Global Ecol Biogeog 12: 483–498.
3. Harvey PH, Clutton-Brock TH (1985) [Life history variation in primates](http://evolve.zoo.ox.ac.uk/publications.html?id=81) Evolution 39: 559–581.
4. Freckleton RP, Harvey PH, Pagel M (2002) Phylogenetic analysis and comparative data: a test and review of evidence. Am Nat 160: 712-726.
5. Sibley CG, Ahlquist JE (1990) Phylogeny and classification of birds. New Haven: Yale University Press.

**Supplementary Table 2.** Median range area and latitude by hemisphere. Correlation coefficients are shown for cells north and south of the equator for: a) all species using all cells and b) species whose midpoints fall within 1 degree latitudinal bands. Significant (* p < 0.05, ** p < 0.01, *** p < 0.001, **** p <<0.0001) correlations indicating increases in range size with latitude are shown in bold.

|  | a) All cells | | | | b) Midpoint species | | | |
| --- | --- | --- | --- | --- | --- | --- | --- | --- |
| Biogeographic | South | | North | | South | | North | |
| Region | r | n | r | n | r | n | r | n |
| Global | -0.29^****^ | 4903 | **0.42^****^** | 12964 | -0.03 | 59 | **0.58^****^** | 67 |
| Australasia | **0.43^****^** | 1452 | **0.48^**^** | 29 | -0.07 | 55 | -0.84 | 4 |
| Antarctica | **0.17^*^** | 213 | --- | --- | **0.71^*^** | 10 | -- | -- |
| Afrotropics | 0.02 | 1184 | **0.63^****^** | 1380 | -0.02 | 38 | -0.52^**^ | 26 |
| Indomalaysia | **0.36^****^** | 138 | **0.54^****^** | 1147 | -0.93^****^ | 12 | -0.04 | 35 |
| Nearctic | --- | --- | **0.26^****^** | 2979 | -- | -- | 0.23 | 48 |
| Neotropics | -0.57^****^ | 1742 | -0.46^****^ | 729 | 0.11 | 54 | -0.39^*^ | 28 |
| Oceania | -0.10 | 174 | 0.36^****^ | 114 | -0.33 | 19 | -0.21 | 16 |
| Palearctic | -- | -- | **0.24^*^**^***^ | 6586 | -- | -- | **0.40^**^** | 54 |
